# Mental Violence: The COVID-19 Nightmare

**DOI:** 10.3389/fpsyt.2020.579289

**Published:** 2020-10-30

**Authors:** Fernanda Cristina Coelho Musse, Laura de Siqueira Castro, Ksdy Maiara Moura Sousa, Thiago Fuentes Mestre, Camila De Masi Teixeira, Sandra Marisa Pelloso, Dalva Poyares, Maria Dalva de Barros Carvalho

**Affiliations:** ^1^Faculty of Medicine, Maringá State University, Maringá, Brazil; ^2^Faculty of Medicine, UniCesumar University, Maringá, Brazil; ^3^Department of Medicine, Universidade Federal de São Paulo, São Paulo, Brazil; ^4^Department of Psychobiology, Universidade Federal de São Paulo, São Paulo, Brazil; ^5^Faculty of Medicine, Uningá University, Maringá, Brazil

**Keywords:** pandemic (COVID-19), dreams, mental health, health behaviors, healthcare personnel

## Abstract

The year 2020 has generated profound changes in personal and working relations, and in dreams of millions of people worldwide. The aim of this study was to investigate the frequency and content of nightmares during the COVID-19 pandemic in Brazil, evaluating its associations with sociodemographic, occupational, and clinical factors. Cross-sectional exploratory study, including 1,057 participants who responded to an online survey about mental violence and nightmares during the pandemic, between May 25 and June 1, 2020. A descriptive analysis of the results was done to obtain frequency tables. McNemar's non-parametric test was used to compare the frequency of nightmares before and after the pandemic, and logistic regression models, to identify factors most strongly associated with the pandemic nightmares. Participants were from 21 Brazilian states, with a mean age of 38 ± 14 years, and 78% women. Half of them (*n* = 529) reported at least one nightmare episode during the pandemic, and 32.9% (*n* = 348) described a pandemic content. There was nearly a 3-fold increase in the occurrence of nightmares “once a week or more” during the pandemic, 9% before vs. 25% after. Prior psychiatric care, suicidal ideation, sleep medication, increased pandemic alcohol consumption, perceiving high risk of contamination, being woman, and of younger age were factors associated with having nightmares during the pandemic. Prior psychiatric care, sleep medication, and age remained significant after excluding participants without nightmares and comparing between individuals with and without a pandemic content. We conclude the COVID-19 pandemic has affected people's dreams. The increase in the frequency of nightmares, their pandemic content, and association with previous conditions are a concerning public mental health issue and should be taken into consideration by authorities and policy makers.

## Introduction

Nightmares are REM parasomnias (1) defined as repeated episodes of dysphoric and well-remembered dreams, which generally involve fight to survive or for physical integrity (2). Although nightmare content differs between cultures, it generally includes attempts to escape an imminent threat, often replicating menacing situations (2, 3). The year 2020 is being marked by the expansion of the “Coronavirus Disease 2019” (COVID-19) pandemic, a new disease with high potential for transmissibility and morbidity (4), forcing many countries to implement extreme measures to restrain its dissemination. One of these global strategies is the establishment of the lockdown (5–8), which is generating profound changes on personal and working relations of millions of people—beyond a health crisis, a real economic threat, particularly in Brazil, one of the five largest countries worldwide in both geographical and populational sizes.

We believe our multiculturalism and diversity reflect on our current state of disagreement regarding measures to restrain the pandemic, and perhaps amplify this global context that has already caused suffering and fear to too many. The imminent threat of being contaminated by the unknown virus is real, particularly to those closely exposed to it, as is the case of healthcare professionals; besides the grief of hundreds of families who have lost their beloved ones (9–16). One could name the COVID-19 pandemic as a “collective mental violence,” involving great emotional impact and a context of potential “collective trauma” of global extension. No precedent data addresses behaviors or mechanisms involved in the metabolization of negative feelings in such a scale of global collectivity, as well as how they might reflect on people's dreams and nightmares.

Most theories dedicated to explaining the origin of dreams and, therefore, nightmares, argue in favor of episodic and autobiographical memory participation, although this is not a consensus (3, 17–21). Since the beginning of the twentieth century, authors analyze dream content as a response to waking experiences (17, 18). Levin and Nielsen (17), for instance, propose that a dysfunction in affective processes leads to conditioning and to repetition of fear memories, producing nightmares through neural networks associated to limbic (hyperactivated) and prefrontal (hypoactivated) areas (17). This becomes more evident among individuals with post-traumatic stress disorder, such as those experiencing periods of war or torture, whom recurrently report repeated nightmares” (11, 22, 23).

We believe that the experience of a pandemic as COVID-19 may be generating a series of potentially traumatic memories. Reasons are countless, the speed in which real time information travels the globe, their morbid content, the consequences and severity of this new disease without treatment, added to the experience of physical distancing, economic impact, interrupted education, changes in working and familial relations, in ways we obtain food, among others. These memories are in the making and they can modify the occurrence of mental disorders in several ways (21, 22). For many of these disorders, their first manifestation is trouble sleeping, particularly nightmares. This becomes even more important when we analyze current evidence demonstrating an association between bad dreams and an increase in suicide and self-injury (24–28); beyond the development of medical diseases (29, 30).

We hypothesize that social distancing and the perception of risk of acquiring COVID-19 impact on the occurrence of nightmares and how they associate to health-risk behaviors and suicidal ideation during this unprecedented moment in time. In order to provide current data to healthcare organizations and policy makers, considering the need for innovative psychosocial interventions in the near future, and stressing the relevance of such threats to public mental health, we designed an online survey aimed at identifying changes in the frequency and content of nightmares and their associated factors.

## Methods

This study was registered on Plataforma Brazil, under the identification number 31799220.4.0000.0104 and approved by the Ethics and Research Committee of Universidade Estadual de Maringá—Paraná—Brazil, approval number 4.045.034, on May 22nd, 2020.

### Participants and Procedures

Participants were invited to complete an online survey entitled “Mental Violence: the COVID nightmare.” Link access to the structured survey, along with an informed consent form, was spread through social media, for 7 days, between May 25th and June 1st, 2020. Initially, the research access link was launched for groups of family members, students, Church members, multidisciplinary health groups, teachers, groups of engineers, artists, parents from nursery schools, and several other groups asked to replicate the link through Facebook and other social media. The survey included questions about nightmares, sociodemographic characteristics, occupational status, health-risk behaviors, and clinical antecedents. Participation was voluntary and anonymous, via an online Google Form. Individuals could participate and invite others in an active way, by individual or collective call (transmission groups).

### Study Design and Instruments

This is an exploratory cross-sectional study including a structured survey elaborated by the researchers, focused on determining the occurrence of nightmares and related events before (until December 2019) and during the pandemic (after January 2020 onwards), analyzing associations with risk perception and self-protection measures.

The survey was structured with the following sections: (1) sociodemographic data—age, gender, ethnicity, marital status, state of residence, and religion; (2) occupational status and exposure to the COVID-19—professional category, occupation, exposure and threat related to coronavirus, as well as social isolation and the use of facial mask; (3) personal and clinical antecedents—health-risk behaviors, sleep, and previous medical and psychiatric conditions, including suicidal ideation related to the pandemic; and (4) nightmares assessment—frequency of nightmares before and during the pandemic (“no nightmares,” “less than once a week,” and “once a week or more”), along with specific content related to the pandemic.

### Definition of Pandemic and Non-pandemic Nightmares

We used two questions to define pandemic and non-pandemic nightmares, based on the literature (31). The first question was close ended and included 10 pre-categorized items based on previously described nightmare contents (violence/aggression, venomous animals, screaming, or seeing blood), as well as on pandemic related situations, such as dying, people dying, or having relatives dying from COVID-19, difficulty breathing, lung failure, and other symptoms. The second question was open-ended and asked for brief examples of nightmares during the pandemic. The answers were analyzed and grouped according to the presence or absence of elements related to the pandemic context, after a series of team discussions.

Pandemic nightmares included contents such as: “being confined and not being able to see anyone,” unexpected and premature deaths, being contaminated or having a family member contaminated, “being chased by infected people,” suffocation or respiratory symptoms, “forgetting to use individual protection equipment,” facing conflicts with medical decisions and inpatients, having COVID-19 symptoms (such as anosmia, loss of taste), hospitalization because of COVID-19, funerals, financial struggle because of the pandemic, “noise from fans and alarms in the ICU,” among others.

Non-pandemic nightmares included: being chased, physical aggression, screaming, family fights, being haunted, extraterrestrials, childhood nightmares, miscarriage, work related conflicts, ghosts, conflicts with family and friends, “being alone and looking for a destiny and not finding it,” having a house destroyed, accidents, failing tests or exams, assaults, robberies, getting lost in strange places, among others.

### Independent Variables

Independent variables included health-risk behaviors, clinical antecedents, and COVID-19 related variables. Participants were asked whether they were following social distancing and self-protection measures, for how long, whether they needed to isolate from family members due to risk of contamination, or for interacting with coworkers with a high risk of exposure to other infected individuals. Usual frequency and amount of alcohol consumed before the pandemic, as well as changes in consumption during the pandemic were also assessed, along with average sleep duration in the past month, use of sleep medication, and previous psychiatric care for insomnia, depression, or anxiety. We also asked whether participants were having suicidal ideation (or a desire to die) during the pandemic.

### Statistical Analysis

Descriptive statistics include absolute frequencies and percentages for categorical variables and means with standard deviation for continuous variables. To compare proportions and test association between groups we used the Chi-square test. To compare the proportion of nightmares before and during the pandemic we performed the McNemar's non-parametric test (32), used for comparison of paired samples. We used logistic regression models to evaluate the strength of association between the independent variables and the occurrence of nightmares during the pandemic. Analyses were performed with IBM SPSS Statistics, version 25 (33).

## Results

### Sample's Characteristics

A total of 1,057 participants from 21 Brazilian states, with a mean age of 38.1 ± 13.7 years (ranging 18–79) and 78% women, responded the questionnaire and were included in this study. Most were white (77%), married (45%), from either the South (55%) or Southeast (36%) regions in Brazil, and declared being Catholic (42%). Over 1/3 of the respondents were healthcare professionals (38%). We observed nearly a 3-fold increase in the frequency of nightmares during the pandemic. Whilst 8.9% (*n* = 94) of the participants had nightmares more than once a week until before the pandemic, during the outbreak this number raised to 25.5% (*n* = 270). Table 1 shows sample characteristics.

**Table 1:** Sample's characteristics (*N* = 1,057).

		***N* (%)**
Gender	Women	829 (78.4)
	Men	226 (21.4)
	Not binary	2 (0.2)
Age	<20	80 (7.6)
	21–29	261 (24.7)
	30–39	278 (26.3)
	40–49	194 (18.4)
	50–59	148 (14.0)
	60>	85 (8.0)
	Not declared	11 (1.0)
Ethnicity	White	817 (77.3)
	Brown	155 (14.7)
	Black	39 (3.7)
	Other	46 (4.4)
Marital Status	Single	406 (38.4)
	Married	477 (45.1)
	Living together	87 (8.2)
	Other	87 (8.2)
Region of Brazil	South	583 (55.2)
	Southeast	379 (35.9)
	Midwest	40 (3.8)
	North	16 (1.5)
	Northeast	38 (3.6)
Religion	No religion	259 (24.5)
	Catholic	446 (42.2)
	Evangelical	167 (15.8)
	Kardecism	127 (12)
	Afro-Brazilian traditions	13 (1.2)
	Other	45 (4.3)
Healthcare professional		409 (38.7)
Nightmares before the pandemic	Never or rarely	367 (34.7)
	Less than once a week	596 (56.4)
	Once a week or more	94 (8.9)
Nightmares during the pandemic	None	528 (50.0)
	Less than once a week	259 (24.5)
	Once a week or more	270 (25.5)
Pandemic nightmares	No report of nightmare	536^*^ (50.7)
	Pandemic content	348 (32.9)
	Non-pandemic nightmare	173 (16.4)

* *Eight participants reported having nightmares during the pandemic but did not describe an example of such nightmares*.

In Table 2, we show associations between sample characteristics and groups defined by the frequency of nightmares during the pandemic. Women, younger, and single individuals were found to report more nightmares, as well as participants without religious affiliations. We observed no differences in the frequency of nightmares by ethnic groups, regions of Brazil, or occupational status. Of all the participants, 32.9% (*n* = 348) reported nightmares with a pandemic content; 42% having these pandemic nightmares less than once a week and 57% once a week or more. Four participants (1%) answered they had no nightmares during the pandemic but did describe a pandemic nightmare in the open-ended question (Table 1).

**Table 2:** Nightmares during the pandemic by sample's characteristics (*N* = 1,057).

	**None**	**Less than once a week**	**Once a week or more**	**Chi-square (*p*-value)**
	***N* (%)**	***N* (%)**	***N* (%)**	
**Gender**				
Women	380 (45.8)	218 (26.3)	231 (27.9)	26.4 (<0.001)
Men	147 (65)	41 (18.1)	38 (16.8)	
**AGE**				
<20	30 (37.5)	23 (28.7)	27 (33.8)	104.9 (<0.001)
21–29	86 (32.9)	78 (29.9)	97 (37.2)	
30–39	122 (43.9)	76 (27.3)	80 (28.8)	
40–49	120 (61.9)	33 (17)	41 (21.1)	
50–59	96 (64.9)	35 (23.6)	17 (11.5)	
60>	69 (81.2)	11 (12.9)	5 (5.9)	
**MARITAL STATUS**				
Single	166 (40.9)	108 (26.6)	132 (32.5)	57.3 (<0.001)
Married	275 (57.7)	113 (23.7)	89 (18.7)	
Living together	27 (31)	30 (34.5)	30 (34.5)	
Other	60 (69)	8 (9.2)	19 (21.8)	
**RELIGION**				
No religion	100 (38.6)	72 (27.8)	87 (33.6)	35.2 (<0.001)
Catholic	243 (54.5)	113 (25.3)	90 (20.2)	
Evangelical	86 (51.5)	37 (22.2)	44 (26.3)	
Kardecism	76 (59.8)	23 (18.1)	28 (22)	
Afro-Brazilian traditions	2 (15.4)	4 (30.8)	7 (53.8)	
Other	21 (46.7)	10 (22.2)	14 (31.2)	
**HEALTHCARE PROFESSIONAL**				
No	324 (50)	151 (23.3)	173 (26.7)	1.8 (0.395)
Yes	204 (49.9)	108 (26.4)	97 (23.7)	
**NIGHTMARES BEFORE THE PANDEMIC^a^**				
Never or rarely	308 (83.9)	33 (9.0)	26 (7.1)	<0.001
Less than once a week	217 (36.4)	216 (36.2)	163 (27.4)	
Once a week or more	3 (3.2)	10 (10.6)	81 (86.2)	
**CONTENT OF THE NIGHTMARES**				
No reports of nightmare	516 (96.3)	15 (2.8)	5 (0.9)	<0.001
Non-pandemic content	8 (4.6)	98 (56.6)	67 (38.7)	
Pandemic content	4 (1.1)	146 (42.0)	198 (56.9)	

^*^The Chi-square statistic is significant at the 0.05 level.

a *Comparison on the frequency of nightmares before and after the pandemic was tested with the McNemar's test*.

### Health-Risk Behaviors and Clinical Antecedents

Drinking generally over two doses of alcohol once a week or more was associated with more nightmares during the pandemic. Both increasing and decreasing alcohol consumption had an effect in the occurrence of nightmares during the pandemic (Table 3). Respondents who slept fewer hours and were taking sleeping pills reported more frequent nightmares during the pandemic, as well as those with a history of psychiatric disturbances. One-third of the participants with more frequent nightmares during the pandemic reported having experienced suicidal thoughts and ideation (Table 3).

**Table 3:** Nightmares during the pandemic by health-risk behaviors and clinical antecedents (*N* = 1,057).

	**None**	**Less than once a week**	**Once a week or more**		**Chi-square**
	***N* (%)**	***N* (%)**	***N* (%)**	***N* total**	**(*p*-value)**
**SMOKING**					
Never smoked	381 (51)	174 (23.3)	192 (25.7)	747	6.9 (0.328)
Passive smoker	32 (39.5)	22 (27.2)	27 (33.3)	81	
Former smoker >6 mo	75 (51.7)	40 (27.6)	30 (20.7)	145	
Current smoker	40 (47.6)	23 (27.4)	21 (25)	84	
**ALCOHOL FREQUENCY**					
Never/rarely	154 (55.6)	54 (19.5)	69 (24.9)	277	12.9 (0.043)
Occasional	253 (50.2)	134 (26.6)	117 (23.2)	504	
1–4 days/week	110 (43)	68 (26.6)	78 (30.5)	256	
5–7 days/week	11 (55)	3 (15)	6 (30)	20	
**ALCOHOL DOSES**					
No consumption	164 (56.9)	56 (19.4)	68 (23.6)	228	30.5 (<0.001)
1–2 doses	229 (53.5)	110 (25.7)	89 (20.8)	428	
3–4 doses	91 (41.4)	59 (26.8)	70 (31.8)	220	
5–7 doses	28 (32.9)	27 (31.8)	30 (35.3)	85	
8 or more	16 (44.4)	7 (19.4)	13 (36.1)	36	
**ALCOHOL DURING THE PANDEMIC**					
No change	368 (55.8)	144 (21.9)	147 (22.3)	659	26.5 (<0.001)
Drinking less	76 (40.9)	48 (25.8)	62 (33.3)	186	
Drinking more	84 (39.6)	67 (31.6)	61 (28.8)	212	
**REPORTED SLEEP DURATION**					
Less than 4 h	8 (29.6)	5 (18.5)	14 (51.9)	27	38.5 (<0.001)
4–6 h	123 (42.4)	68 (23.4)	99 (34.1)	290	
6–8 h	323 (56.1)	145 (25.2)	108 (18.8)	576	
More than 8 h	74 (45.1)	41 (25)	49 (29.9)	164	
**IN USE OF SLEEP MEDICATION**					
No	457 (53.8)	206 (24.2)	187 (22)	850	34.0 (<0.001)
Yes	71 (34.3)	53 (25.6)	83 (40.1)	207	
**PREVIOUS PSYCHIATRIC CONDITIONS**					
No	319 (64.7)	95 (19.3)	79 (16)	493	83.3 (<0.001)
Yes	209 (37)	164 (29.1)	191 (33.9)	564	
**SUICIDE IDEATION DURING THE PANDEMIC**					
No	501 (54.6)	226 (24.6)	190 (20.7)	917	93.5 (<0.001)
Yes	27 (19.3)	33 (23.6)	80 (57.1)	140	

*Results are based on non-empty rows and columns in each innermost sub-table*.

^*^The Chi-square statistic is significant at the 0.05 level.

### Risk Perception and Self-Protection Measures During COVID-19

Table 4 shows associations between the frequency of nightmares during the pandemic and variables related to COVID-19. “Perceiving high risk of contamination” and “isolation from family members” were associated with more frequent nightmares. Positive COVID-19 diagnosis, judging it dangerous, contacting exposed individuals, following isolation measures, and the use of facial masks were not associated. We also checked whether the frequency of pandemic and non-pandemic nightmares would be different between healthcare professionals and individuals with other occupations, and we observed no effects.

**Table 4:** Nightmares during the pandemic by variables related to the COVID-19 pandemic.

	**None**	**Less than once a week**	**Once a week or more**		**Chi-square (*p*-value)**
	***N* (%)**	***N* (%)**	***N* (%)**	***N* total**	
**COVID-19 POSITIVE**					
Yes	10 (62.5)	2 (12.5)	4 (25)	16	1.4 (0.480)^a^
No	518 (49.8)	257 (24.7)	266 (25.6)	1041	
**JUDGING THE COVID-19 DANGER**					
Little dangerous	14 (63.6)	2 (9.1)	6 (27.3)	22	4.9 (0.294)^a^
Moderately dangerous	144 (53.1)	61 (22.5)	66 (24.4)	271	
Extremely dangerous	370 (48.4)	196 (25.7)	198 (25.9)	764	
**RISK ASSESSMENT OF CONTRACTING COVID-19**					
None/Low risk	118 (55.1)	46 (21.5)	50 (23.4)	214	16.0 (0.003)^*^
Moderate risk	283 (52.6)	135 (25.1)	120 (22.3)	538	
High risk	127 (41.6)	78 (25.6)	100 (32.8)	305	
**CONTACT WITH PEOPLE WHO MAY BE INFECTED**					
Yes	179 (50.1)	90 (25.2)	88 (24.6)	357	0.2 (0.868)
No	349 (49.9)	169 (24.1)	182 (26)	700	
**FULFILLING SOCIAL ISOLATION MEASURES**					
Yes	406 (49.9)	203 (25)	204 (25.1)	813	0.5 (0.743)
No	122 (50)	56 (23)	66 (27)	244	
**ISOLATION FROM FAMILY MEMBERS**					
Yes	202 (48.6)	92 (22.1)	122 (29.3)	416	5.7 (0.058)
No	326 (50.9)	167 (26.1)	148 (23.1)	641	
**USE OF MASK EVERY TIME YOU LEAVE THE HOUSE IN THE LAST 4 WEEKS**					
Yes	503 (50.3)	246 (24.6)	251 (25.1)	1000	0.4 (0.236)^a^
I don't leave home	14 (38.9)	7 (19.4)	15 (41.7)	36	
No	11 (52.4)	6 (28.6)	4 (19)	21	

*Results are based on non-empty rows and columns in each innermost sub-table*.

* *The Chi-square statistic is significant at the 0.05 level*.

a *More than 33% of cells in this sub-table have expected cell counts <5. Chi-square results may be invalid*.

In the multiple regression analysis, we applied the Backward stepwise selection method to retain variables and found the following independent risk factors for the occurrence of nightmares during the pandemic, independently of their frequency: being woman, of younger age, having previous psychiatric conditions, use of sleep medication, perceiving a high risk of contracting COVID-19, increased alcohol consumption during the pandemic, and suicidal ideation. The later with the strongest effect, that is, individuals reporting suicidal ideation during the pandemic were almost 3 times more likely to be experiencing nightmares. In a second model, we excluded individuals without nightmares and compared between those reporting or not nightmares with a pandemic content. Previous psychiatric conditions, the use of sleep medication, and younger ages were the variables that remained significant and with independent effects on pandemic nightmares (Table 5).

**Table 5:** Independent factors associated with the occurrence of nightmares and pandemic nightmares.

	**Model 1 (*****n*** **=** **1,057)**		**Model 2 (*****n*** **=** **521)**	
	**Having or not nightmares during the pandemic**		**Having or not nightmares with a pandemic content**	
	**OR value**	***p*-value**	**OR value**	***p*-value**
**Suicidal ideation**	2.8 (1.7–4.7)	<0.001		
**Previous psychiatric conditions**	2.3 (1.7–3.0)	<0.001	1.6 (1.0–2.4)	0.019
**Alcohol during the pandemic**				
No change	–	0.072		
Less consumption	1.3 (0.9–1.9)	0.133		
More consumption	1.4 (1.0–2.1)	0.042		
**Perception of risk of contamination**				
Low risk	–	0.019		
Moderate risk	1.0 (0.7–1.5)	0.746		
High risk	1.6 (1.0–2.4)	0.019		
**Use of sleep medication**	1.9 (1.3–2.8)	<0.001	1.6 (1.0–2.7)	0.033
**Women**	1.9 (1.4–2.8)	<0.001		
**Age**	0.9 (0.9–1.0)	<0.001	0.9 (0.9–1.0)	<0.001
**Constant**	1.109	0.745	4.192	<0.001

*Logistic regression models using Backward stepwise variable selection method. Variables entered on step 1 of both models were: Previous psychiatric conditions, suicide ideation during the pandemic, sleep duration, sleep medication, occupational status, smoking, frequency of alcohol consumption, change in alcohol consumption during the pandemic, following social distance measures, use of facial mask, positive COVID-19 diagnosis, perceived danger of the COVID-19, perceived risk of contracting COVID-19, contact with exposed individuals, isolation from family members, gender, and age*.

*Model 1 stopped at the 11th step, whereas model 2 at the 15th step*.

## Discussion

This is one of the first studies to analyze nightmares in the context of the COVID-19 pandemic. Compared to the frequency of nightmares seen before the pandemic, we observed nearly a 3-fold increase in the number of participants reporting nightmares “once a week or more” during the pandemic. Over 2/3 of these participants also described nightmares with a pandemic content. These findings are supported by previous theoretical conceptualizations reporting dreams and nightmares as adaptative biological responses with an adjustment function (18).

According to epidemiological data, ~6–16% of the general population report having nightmares at least once a week (3, 34, 35). The prevalence of nightmares is generally more frequent among younger individuals, particularly children and adolescents as compared to adults (1, 2, 23, 35). Women are more likely to complaint of nightmares, also individuals with other psychiatric conditions, such as those with post-traumatic stress disorders (2, 22, 23).

Several theoretical studies indicate that not all recent daytime experiences will turn into oniric experiences, but rather, those with emotional content (36, 37). The significant high frequency of nightmares may reflect the anxiogenic context experienced during the unprecedented pandemic, emphasizing the status of “mental violence” or “collective trauma.” Nightmares are REM parasomnias; which role seems to be linked to consolidation of memories processes and emotional memories (38).

In this context, with a significant proportion of participants reporting nightmares during the pandemic, and most of them have pandemic content, this becomes particularly relevant given the anxiogenic new condition, in which people tend to have negative interpretations of reality. The exposure to pandemic-related stressors and the emotional content of daily memories could corroborate to explain some aspects of nightmares disorders and REM parasomnia (39, 40). Thus, experiences with intense emotional content, whether positive or negative, would be more easily stored and, consequently, processed during REM sleep, by the activation of cortical regions related to recognition and emotion confrontation, such as amygdala, hippocampus, medial prefrontal cortices and the anterior cingulate gyrus (38, 41).

As previously noted, dysfunctional affective processes may lead to a repeating reprocessing of fear memories (17). Neural networks associated with limbic areas (hyperactivated) and prefrontal (hypoactivated) are related to this repetitive activation of fear memories, which would generate the nightmares, and whose frequency increases when the emotional charge or stress level elevate (17, 35). This would happen in patients with mental disorders such as anxiety, depression and post-traumatic stress disorder (PTSD) (17, 35). Indeed, having previous history of psychiatric disorder increased the risk of nightmares in our study. We have also found that women are more likely to have nightmares than men and that the frequency of nightmares decreases with age. It might be expected, since anxiety, depression and insomnia are also more prevalent among women (42). With regard to age, the prevalence of nightmares is higher among children and younger individuals and tend to lower with aging (1, 2, 23, 35, 43).

We have not found significant difference between the frequency of pandemic and non-pandemic nightmares, when comparing the results of health professionals to that from the general population, although we found a greater chance of nightmares among those who had a perceived increased risk of contamination. Interestingly, when pandemic-related content nightmares were regarded, only age, previous psychiatry condition, and taking sleeping pills.

Another possible explanation for this result, may rely on the fact that even though the health professionals spend more time in contact with the new COVID-19 disease reality, the general population is over-exposed to real-time information of great emotional impact, especially through the media and social networks. Gao et al. (44) made an association between the prevalence of mental health issues and the social media exposure during the pandemic, in a sample of 4,872 participants, demonstrating the high prevalence of mental health issues associated to the “infodemic” during the COVID-19 outbreak.

Another relevant result refers to the significant association between the frequency of nightmares during the pandemic and the 2.6-fold increase in the chance of suicidal ideation. Tae et al. (45) reported, with a sample of 909 adults the correlations between sleeping problems and suicidal ideation, associated or not with depressive disorders, finding out that more than 94% of the participants with suicidal ideation also had sleeping problems. However, another study demonstrated that insomnia and nightmares are associated with an elevated suicide risk as well as suicidal thoughts and behaviors among a sample of young adults (24).

Regression results also showed a dose-response association between alcohol consumption and the presence of nightmares. This is in agreement with previous report that compared a group of 37 patients with alcohol addiction to a group of 35 non-addicted patients, and found that the quality of sleep was lower among the alcohol addicted individuals and the subjective experience of dreams was more negatively toned in this group, improving after 4 weeks of abstinence (46). Similar result was found among sleeping pills users. Drugs acting on GABAergic receptors, as well as with sedative hypnotic effects are likely to induce nightmares in some patients (47).

Potential limitations of this study refer to selection bias. We describe a non-probabilistic sample including mostly women who responded to an online survey about nightmares during the pandemic. It is possible that a larger proportion of individuals suffering from nightmares responded and men are underrepresented. Nevertheless, participants were from 21 out of 26 Brazilian states, half had no nightmare complaint whatsoever, and the prevalence of frequent nightmares before the pandemic—occurring at least once a week and as described in diagnostic manuals—was similar to that described by epidemiological studies (3, 34, 35). Moreover, we were particularly interested in seeing the frequency of pandemic contents among individuals with and without previous nightmare complaints. The other potential limitation is that nightmare frequency was assessed at a single point in time, and therefore relied on participant's long-term memory from the pre-pandemic period. People might be more likely to recall nightmares that happened in the recent past rather than nightmares that happened a while ago. As aforementioned, however, nightmare frequency reported for the pre-pandemic period is similar to general nightmare frequency reported in the literature. Future research could evaluate whether nightmare frequency changes if measured with a dream-log that participants fill in everyday for a period.

In summary, our findings suggest the COVID-19 pandemic is significantly affecting people's lives and dreams, what is evidenced by the significant increase in the frequency of nightmares and in association with suicidal ideation, as well as with changes in health-risk behaviors. High frequency of nightmares whether related or not to pandemic contents, should be inquired by healthcare professionals, as it is associated with significant mental health morbidity.

## Data Availability Statement

The raw data supporting the conclusions of this article will be made available by the authors, without undue reservation.

## Author Contributions

FM, MC, LC, DP, and CT wrote the manuscript. FM conducted the analyses supervised by MC. LC conducted the analyses. All authors contributed to the study design, critically reviewed the manuscript, and approved the final manuscript.

## Conflict of Interest

The authors declare that the research was conducted in the absence of any commercial or financial relationships that could be construed as a potential conflict of interest.
